# When Is Humiliation More Intense? The Role of Audience Laughter and Threats to the Self

**DOI:** 10.3389/fpsyg.2017.00495

**Published:** 2017-04-20

**Authors:** Liesbeth Mann, Allard R. Feddes, Anne Leiser, Bertjan Doosje, Agneta H. Fischer

**Affiliations:** ^1^Department of Social Psychology, University of AmsterdamAmsterdam, Netherlands; ^2^Department of Social Sciences and Humanities, Jacobs University BremenBremen, Germany

**Keywords:** humiliation, audience laughter, self, identity, values, social support

## Abstract

In personal accounts, humiliation is often reported as a very intense, painful, negative emotion. We report two scenario studies in which we explored two factors that may contribute to the intense character of humiliation: (1) unwanted, negative public exposure, and (2) a threat to central aspects of one's identity. Study 1 (*N* = 115) assessed emotional reactions to a public insult when an audience responded with either laughter or not and when someone from the audience offered support after the insult or no support was offered. Results showed that the intensity of humiliation increased when people laughed after the insult. However, support offered after the insult had no effect on reported humiliation. Study 2 (*N* = 99) focused on threats to different self-related values and showed stronger reports of humiliation when central self-related values were threatened than when less central self-related values were threatened. Study 2 also replicated the audience-effect from Study 1, but only when central self-related values were threatened and not when less central self-related values were threatened. Limitations of these studies (e.g., the use of scenarios) and potential avenues for future research, such as the (long-term) consequences of humiliation and humiliation in the context of social media, are discussed.

## Introduction

*I fell in the dirt. The pizza fell on top of me. The Diet Pepsi tipped over and glugged out all over my dress. The table fell on top of the Pepsi on top of the pizza on top of me. The napkin fluttered away. EVERYONE LOOKED AT ME*.

*[…] I'm sure nobody else in that dusty press paddock remembers the time some rando[m] fat chick fell down. […] But I WILL NEVER, EVER FORGET IT. That's the thing about humiliation—it sticks with you. It becomes a part of you. Because it's not an external emotion, like anger, it's internal. It's losing your grip on the image of yourself you're trying so desperately to control and project. It tears down the curtain. It undermines who you think you are as a person, and that's frightening (West*, *2014**)*.

Humiliation seems to be a “darker” and more pervasive emotional experience than many other emotions. The quote above illustrates humiliation's intense and painful character. This intensity is, for example, reflected in a tendency for humiliating experiences to remain vivid in the minds of victims, regardless of the amount of time that has passed (Klein, 1991). In line with this, some recent empirical evidence on the neural processing of emotions has showed that humiliation is a very intense emotion, more so than related negative emotions such as shame and anger (Otten and Jonas, 2014).

In the present article, we follow Hartling and Luchetta's (1999, p. 264) definition of humiliation as “the deep dysphoric feeling associated with being, or perceiving oneself as being unjustly degraded, ridiculed, or put down—in particular, one's identity has been demeaned or devalued.” We further consider humiliation a unique mix of emotions as it has been associated with shame and avoidance tendencies, but also with anger and tendencies to attack and take revenge (e.g., Elison and Harter, 2007; Mann et al., 2016; Mann et al., manuscript in preparation).

Our primary focus in the present studies is on explaining why the experience of humiliation is often reported to be so intense. In particular, we examine two potential reasons. The first is derogative, public exposure. This is especially the case with a laughing audience, because laughter in a negative situation is generally perceived as derogatory, and amplifies the threat to one's identity. A second reason for humiliation's intensity may be found in the specific target of the humiliation, in other words, which aspects of oneself are being threatened: central, stable, and unique elements vs. less central, changeable, and situation-dependent elements of one's identity. A threat to central elements of the self is likely to be associated with more intense feelings of humiliation than a threat to less central elements of the self. We examine these two factors in two scenario studies, in which we manipulated the response of an audience during an imaginary humiliating episode (Study 1), and examined several humiliating situations that vary in the extent to which they target central or less central aspects of the self (Study 2).

### Humiliation and negative public exposure

Perhaps not surprisingly, humiliation—especially when experienced frequently—has been associated with a host of psychological, relational, and societal problems, as well as with clinical disorders such as low self-esteem, depression, general anxiety disorder, suicidal intentions, homicide and (domestic) violence (e.g., Klein, 1991; Gilbert, 1997; Hartling and Luchetta, 1999; Farmer and McGuffin, 2003; Kendler et al., 2003; Leary et al., 2003; Elison and Harter, 2007; Torres and Bergner, 2010; Walker and Knauer, 2011; Harter, 2012; Collazzoni et al., 2014, 2015). One often cited example of the destructive potential of humiliation is the phenomenon of *school-shootings*, the (attempted) mass killing and injuring of students and teachers at a school or university by one or more students of that institution. The shootings are (at least partly) considered to be the result of the frequent humiliation that has been experienced by these perpetrators (Leary et al., 2003; Elison and Harter, 2007; Torres and Bergner, 2010; Harter, 2012).

But what causes people to feel humiliated? In general, the experience of humiliation can result from being the center of negative attention, as is the case when being teased, harassed, ridiculed or put down (Elison and Harter, 2007; Harter, 2012), but it can also follow from being neglected, excluded or ostracized (Hartling, 2007; Veldhuis et al., 2014). These acts of humiliation (bullying, excluding) can be triggered by a strong norm violation or transgression by the subject, but also merely by being “different” or judged by others as inadequate (Harter, 2012).

Humiliation is often associated with shame (e.g., Lewis, 1971, 1992; Miller, 1993; Hartling and Luchetta, 1999; Lindner, 2009). Indeed, both emotions are negative and concern the self (Zavaleta Reyles, 2007) and both are the result of global attributions; i.e., a focus on the total or core self, rather than on specific aspects of the self, such as is the case with guilt (Lewis, 1995). Furthermore, they both arouse a wish to hide from others (Harter, 2012). However, humiliation and shame differ in that shame entails a judgment or criticism on the self by the *self*, whereas humiliation entails a judgment or criticism on the self by *another*. This implies an appraisal of unfairness, which is a central aspect of humiliation (Klein, 1991; Hartling and Luchetta, 1999; Jackson, 2000; Elison and Harter, 2007; Combs et al., 2010; Torres and Bergner, 2010; Harter, 2012).

Humiliation is also connected to embarrassment. Elison and Harter (2007) report some overlap between these emotions, but they and others also note important differences, especially in terms of the perception of others' hostile intent (e.g., Elison and Harter, 2007; Combs et al., 2010; Harter, 2012). This hostile intent drives the association between humiliation and anger or even rage. Although anger and humiliation clearly differ, the appraisals of unfairness and perceived hostile intent show the similarities between humiliation, anger, aggression, and a desire for revenge (e.g., Smith et al., 2002; Elison and Harter, 2007; Combs et al., 2010; Leidner et al., 2012; Fernández et al., 2015).

A key element in the experienced intensity of humiliation seems to be the presence of other people witnessing the humiliating event (e.g., Klein, 1991; Hartling and Luchetta, 1999; Elison and Harter, 2007). Smith et al. (2002) argue that “public exposure of any sort of behavior, and the evaluative implications of public scrutiny, may be an especially powerful ingredient of the socially constructed self” (p. 146). Supporting this argument, there is evidence that public exposure of a wrongdoing leads to stronger reports of shame, humiliation, and embarrassment than when the wrongdoing remains private (see also Combs et al., 2010; Fernández et al., 2015). Elison and Harter (2007) further showed that the presence of an audience displaying hostile intent is judged as prototypical for humiliation. Thus, although humiliation can be felt without an audience being present, we argue that the most typical and intense instances of humiliation are those in which others are present, showing hostile intent.

There are various ways of signaling hostile intent. In the present research we focus on laughter in response to a negative event. Although laughter often has a clear positive and prosocial function (see e.g., Sauter et al., 2010; Scott et al., 2014), it can also be a signal of negative emotions (see also Niedenthal et al., 2010). For example, the emotion of contempt is often accompanied by a smile, referred to as the unilateral lip curl (e.g., Ekman and Friesen, 1986; Wagner, 2000; Fischer and Giner-Sorolla, 2016) and can be expressed with scornful and derisive laughter (Ruch and Proyer, 2008). *Schadenfreude*—the enjoyment of others' misfortune (van Dijk et al., 2006)—is another negative emotion associated with laughter (see Ruch and Proyer, 2008). In such instances, laughter is not friendly or supportive, but rather derogative and implies that we *laugh at* rather than *with* someone. In the case of humiliation, we therefore expect that in situations where a person encounters hostile remarks, others' laughter is a clear signal of degradation or derision.

### The specific target of humiliation

A second explanation for the painfulness of humiliation is related to the target of the humiliative act. We usually do not feel humiliated when someone laughs at us because of our opinion about cats, unless someone defines herself as a fanatic cat lover. We therefore make a distinction between important and central parts of the self, referring to preferable traits that are considered stable and relatively unchangeable, vs. more malleable and situation-dependent parts of the self. These latter aspects can be adjusted and changed, and we argue that these traits should therefore be less prone to humiliative acts. For example, specific preferences or attitudes could be denied or downgraded in response to a humiliating incident. Humiliation has been described as “an invasion of the self” (Klein, 1991, p. 98), and we therefore assume that a humiliating episode should entail a threat to central and stable aspects of one's identity.

We based the operationalization of central and stable vs. more flexible and malleable aspects of the self on research into people's values. Values are concepts or beliefs about desirable end states or behavior (Schwartz and Bilsky, 1990) and thus, they reflect what is important in people's lives. However, some values, the “high-priority values” (e.g., Schwartz and Bilsky, 1990), are more important than others, because they are more central to the self-concept. In Western culture, autonomous values, such as independence, openness, and originality are considered more defining of and important to the self (e.g., Markus and Kitayama, 1991), whereas social-relational values such as respect for tradition and family are less central and more changeable. In line with this idea, we predict that when a humiliating act consists of a threat to autonomous self-related values, people from Western cultures feel more humiliated than when the humiliating act concerns a threat to social-relational self-related values.

### The current research

Our first set of hypotheses relate to the role of an audience. We hypothesize that the presence of a laughing audience during a humiliating episode intensifies feelings of humiliation. In addition, we examine the effect of a contrasting audience response, namely the target of humiliation being socially supported by an individual from the audience, which may reduce humiliation. Social support has, to our knowledge, not been experimentally studied in the specific context of humiliation. However, research on classroom bullying showed that so-called *peer support systems* (e.g., Naylor and Cowie, 1999; Cowie and Hutson, 2005)—trained youngsters who offer friendship and support to victims of bullying, and promote a pro-social atmosphere in and around the classroom—reduce the negative impact of bullying on the victims (Cowie and Hutson, 2005). In line with this, we argue that social support after a humiliating event may help the victim to cope with the experience and thus reduces feelings of humiliation.

Secondly, we focus on the type of threat that the humiliating situation entails and hypothesize that, in our Western sample, a threat to stable aspects of one's identity (e.g., autonomous values) rather than to more malleable aspects of the self (e.g., social-relational values), in a humiliating episode increases the relevance of the situation, which may make this situation more humiliating.

Finally, based on Elison and Harter's (2007) conclusion that the dynamics of humiliation apply to both genders, we have no reason to expect gender differences. However, previous studies on gender differences in subjective emotions have shown more intense reports of emotions by women (Fischer and Evers, 2013), and one study showed higher reports of humiliation by women than by men (Hartling and Luchetta, 1999). Furthermore, there may be gender differences in the importance attributed to certain self-related values (e.g., Schwartz and Rubel, 2005). For example, women may regard social-relational values as more important than men do^1^. Thus, we control for gender in our studies.

We report two studies focusing on these ideas. In Study 1, we examine whether a description of a public insult in the presence of an audience increases reports of humiliation when the audience laughs compared to when there is no audience response (Hypothesis 1). We also expect that including social support from someone witnessing the episode in such a description decreases reports of humiliation (Hypothesis 2). In Study 2, we examine whether humiliation is stronger when central and stable aspects of the self are threatened than when less central and changeable aspects of the self are threatened (Hypothesis 3). We also aimed to replicate the potential effect of audience laughter on reported humiliation. We confirm that we report all data exclusions, all measures and all manipulations in the two studies.

## Study 1

In Study 1 we manipulated the response of an audience during a hypothetical humiliating episode. We constructed a scenario that describes a person being accused of not being able to give their honest and open opinion. This happens in the presence of others (the audience) who either laugh after the insult or show no response. Participants were requested to imagine themselves as the protagonist in the story and to indicate their expected emotional reaction. We predicted that reports of humiliation would be higher when the audience laughs at the protagonist than when there is no such response (Hypothesis 1). We also expected that support offered after this episode would decrease reported intensity of humiliation (Hypothesis 2).

### Method

#### Participants and procedure

A total of 160 participants took part in this study. Data were collected online by mailing the student mailing list of an English-speaking international university in Germany. Additionally, we collected data (paper and pencil) in and around the University of Amsterdam and via a snowball procedure in the United Kingdom.

As the current study was not focused at testing cultural differences in these situations, we aimed for a culturally homogeneous group of people from Western Europe. However, 28 international students of the German university were born and raised in non-Western, collectivistic countries (i.e., Africa, Latin America, and Asia). For ethical reasons we did not exclude students based on nationality, but we analyzed only the results of participants from Western countries. Importantly, when we included the data of the non-Western participants we found the same patterns of significant results. Data of another 17 participants were excluded for other reasons^2^. Thus, 115 participants remained (72 female, 42 male, 1 gender missing). Their mean age was 27.34 years (*SD* = 10.81, range: 14–64).

Participants read the scenario and completed the questionnaire called “Emotions in Daily Life.” Depending on their country of origin, a Dutch or an English version was presented (see below for translation procedures).

#### Design and scenario

The scenario and questions were first written in Dutch and then translated into English by the researchers. These translations were inspected and where necessary corrected by a native speaker. We used a 2 (Audience Response: No Response vs. Laughter) × 2 (Social Support: No Support vs. Support) between-participants design with Gender as a covariate. Participants were randomly assigned to one of the four conditions. In all conditions the first part of the scenario consisted of the following text:

You are participating in a discussion on politics, hosting people with different backgrounds. At a certain moment the discussion turns to a sensitive subject for some people. The discussion leader happens to be aware that you know a lot about this subject and he asks you a question about it. When you hesitate a little to answer his question, the person who sits next to you says in a sneering tone: “If you are not even able to give an honest and open opinion, then what are you doing here?”

Subsequently, in the Laughter condition the sentence: “Some of the other participants start to laugh,” was added to the text. We used the word “laughter,” because this is a neutral description of a display that can be interpreted both positively (affiliation) and negatively (derision). If “laughter” would increase reported feelings of humiliation, this would be a conservative test, and show that any form of laughter may be interpreted in a negative way in such contexts.

We did not want to check this manipulation in the current study as we feared asking participants about the audience's response would raise suspicion about the aim of the study and evoke unwanted effects. Therefore, the laughter manipulation was checked in a pilot-study (*N* = 158) using the same scenario in which we asked participants to indicate how the audience responded (i.e., “They laughed,” “They did not respond,” “They were angry,” “It was not stated,” “I don't know”). All participants in the Laughter condition indicated that the audience laughed and only 5.1% of participants in the No Response condition indicated that the audience laughed.

In the Support condition the sentence: “Then, another participant tells the person next to you: ‘Don’t act so stupid, have some respect!”' was added. This manipulation was checked in the current study as we could measure support more implicitly and therefore had no reason to expect unwanted effects of this check.

#### Measures

Participants indicated their agreement with statements on a scale ranging from 1 (*not at all*) to 5 (*very much*). Humiliation was measured with the following item: “In this situation I would experience humiliation^3^.” To check our manipulation of social support we asked the following question: “Do you feel the participants were on your side?” (felt support).

### Results

#### Manipulation check

An analysis of variance (ANOVA) was performed with Social Support (No Support vs. Support) and Audience Response (No Response vs. Laughter) as factors and participants' rating of the extent they would feel that the other discussants were on their side (felt support) as dependent variable. There was no main effect of Social Support. However, there was a significant interaction between Audience Response and Social Support, *F*_(1, 111)_ = 14.11, *p* < 0.001, $\eta_{p}^{2}$ = 0.113. When the audience did not react after the insult, there was no difference in felt support in the two conditions (*M*_Support_ = 3.04, *SD* = 0.94; *M*_No Support_ = 3.35, *SD* = 0.63). However, when the audience laughed after the insult, support resulted in higher scores on felt support than when no support was given (*M*_Support_ = 3.29, *SD* = 0.60; *M*_No Support_ = 2.50, *SD* = 0.86). These results suggest that the manipulation of support was partly successful, as the extent to which people indicated to feel support depended on the presence of audience laughter.

#### Main analysis

A univariate analysis of covariance (ANCOVA) with Audience Response (No Response vs. Laughter) and Social Support (No Support vs. Support) as between-subjects factors, Gender as a covariate, and reported humiliation as dependent variable showed a significant main-effect for Audience Response, *F*_(1, 109)_ = 5.77, *p* = 0.018, $\eta_{p}^{2}$ = 0.050. When scenarios included audience laughter, reported humiliation was higher (*M* = 2.84, *SD* = 1.08) than when there was no audience response after the insult (*M* = 2.30, *SD* = 1.20), supporting Hypothesis 1.

Contrary to Hypothesis 2, there was no effect of Social Support on reported humiliation, *F*_(1, 109)_ = 0.03, *p* = 0.865, $\eta_{p}^{2}$ = 0.000. However, in line with our hypothesis, there was a significant negative relation between felt support and humiliation, *r* = −0.21, *p* = 0.025, indicating that the stronger the participants believed that the other discussants were on their side, the less strongly they reported humiliation. There was no significant interaction between Audience Response and Social Support^4^.

Finally, Gender was not a significant covariate, *F*_(1, 109)_ = 1.80, *p* = 0.183, $\eta_{p}^{2}$ = 0.016.

### Discussion

The results of Study 1 support the idea that derogative audience laughter after an insult leads to stronger humiliation compared to the same insult without audience laughter, confirming Hypothesis 1. Contrary to Hypothesis 2, the manipulated social support from someone in the audience did not reduce reports of humiliation after the insult, nor did it reduce reported humiliation after audience laughter. We may explain the absence of an effect of support on humiliation by the fact that receiving social support can actually emphasize one's vulnerable and low status. Moreover, the involvement of others also stresses the public nature of the degradation, which may, in some cases, lead to an unintended *negative* effect of social support (although we did not find such an effect in the current study). We will discuss the role of social support further in the General Discussion.

## Study 2

Study 1 supported Hypothesis 1 that a public insult followed by audience laughter is perceived as more humiliating than when the audience does not react after the insult. In Study 2, we further examined whether an insult would be perceived as especially painful in response to specific types of threats to the self. Previous research has shown that values reflecting one's autonomy and stable personality are a more central part of self-construals of people from Western-European countries than values related to one's connectedness with others (e.g., Markus and Kitayama, 1991). We therefore studied whether in the current (Dutch) sample, an insult would enhance humiliation in particular when one's autonomous self, e.g., one's independence or honesty, is at stake rather than when the social-relational self is threatened.

To this end, we created six scenarios describing a public insult targeted either at the autonomous or at the social-relational self. We asked participants to read all six scenarios and imagine themselves as protagonist. The response of the audience (laughter or no response) was manipulated in the same manner as in Study 1.

### Method

#### Participants and procedure

Participants were 101 students from two universities in Amsterdam. They had different ethnic backgrounds, but Dutch was their mother tongue. Data of two participants were not analyzed because they did not participate in a serious manner. Thus, 99 participants remained. Their mean age was 21.92 (*SD* = 4.41, range: 18–42, 71 female).

Students came to the lab and participated in return for credits. In addition, participants were recruited at the University campus. As a small token of appreciation they were offered a candy bar. Participants read the scenarios and completed the questionnaire called “Social Situations.”

### Scenarios and dependent measures

In all six scenarios the protagonist is insulted by someone on the basis of a self-related value in the presence of other people who either laugh or do not react. Participants were randomly assigned to one of these two conditions (Laughter or no Response). Three scenarios described threats to values related to autonomy (honesty/openness, independence, and originality), and three scenarios described threats to social-relational values (respect for tradition/family, respect for elderly, and helpfulness). These values were selected on the basis of Schwartz's (2006) research on cultural values. For the exact wording of the scenarios, see Appendix A. Before presenting the scenarios, we measured endorsement of the autonomy values (i.e., honesty, independence, and originality) and social-relational values (i.e., respect for elderly, respect for tradition, helpfulness) by asking participants to rate their importance. These questions could be answered on a scale ranging from 1 (*not important at all*) to 7 (*very important*). After each scenario, we measured humiliation with the item: “In this situation I would feel humiliated.” These questions could be answered on a scale ranging from 1 (*not at all*) to 7 (*very much*)^5^.

### Results

#### Endorsement of autonomy- and social-relational values

We created two scales of value endorsement (autonomy and social-relational value endorsement). However, the reliability of these scales was very low (Cronbach's α = 0.20 and 0.58 respectively). Therefore we could not use the scales in further analyses (e.g., to test a mediation of value endorsement). Nevertheless, participants scored higher on the autonomy values scale (*M* = 5.86, *SD* = 0.63) than on the social-relational values scale (*M* = 5.36, *SD* = 0.88), *t*_(98)_ = 5.67, *p* < 0.001, *r* = 0.50. This indicates that participants thought the autonomy values were more important for them than the social-relational values. We took this as evidence that the autonomy values were a more central part of participants' identity than the social-relational values.

#### Main analyses

First, we collapsed scores for humiliation for the three autonomy and the three social-relational scenarios separately^6^. In line with Hypothesis 3, a paired-samples *t*-test showed that participants reported more humiliation after reading scenarios describing threats to autonomous self-related values (*M* = 4.05, *SD* = 1.39) than after reading scenarios describing threats to social-relational self-related values (*M* = 3.31, *SD* = 1.28), *t*_(98)_ = 5.98, *p* < 0.001.

Next, because our research design prevented us from performing a mixed design analysis (with Type of Scenario as within-subjects factor and Audience Response as between-subjects factor), we analyzed the potential effect of Audience Response on humiliation for the autonomy- and social-relational scenarios separately.

#### Autonomy scenarios

We conducted an ANCOVA with Audience Response (No Response vs. Laughter) as between-subjects factor, Gender as a covariate, and reported humiliation as dependent variable. There was a significant effect for Audience Response, *F*_(1, 96)_ = 8.21, *p* = 0.005, $\eta_{p}^{2}$ = 0.079, indicating that participants who read scenarios in which the audience laughed reported stronger humiliation (*M* = 4.38, *SD* = 1.36) than participants who read scenarios without audience laughter (*M* = 3.69, *SD* = 1.35)^7^. Gender was a significant covariate, *F*_(1, 96)_ = 10.88, *p* = 0.001, $\eta_{p}^{2}$ = 0.102.

We also analyzed the effect of Audience Response for each scenario separately (for means and standard deviations see Table 1). A multivariate analysis of covariance MANCOVA with Audience Response (No Response vs. Laughter) as between-subjects factor, Gender as a covariate and reported humiliation for scenarios 1, 2, and 3 (see Appendix A) as dependent variables indicated an overall main effect, Wilks' Lambda = 0.902, *F*_(3, 93)_ = 3.37, *p* = 0.022, $\eta_{p}^{2}$ = 0.098. Univariate ANCOVAs indicated a significant effect for scenario 1, *F*_(1, 95)_ = 9.11, *p* = 0.003, $\eta_{p}^{2}$ = 0.088, and scenario 3, *F*_(1, 95)_ = 5.04, *p* = 0.027, $\eta_{p}^{2}$ = 0.050, and a marginally significant effect for scenario 2, *F*_(1, 95)_ = 3.20, *p* = 0.077, $\eta_{p}^{2}$ = 0.033. For each scenario, participants reported stronger humiliation when the audience laughed than when there was no audience response after the insult, replicating findings from Study 2.

**Table 1:** **Means and standard deviations for humiliation after reading autonomy- or social-relational scenarios, separated for audience response (No Response vs. Laughter), Study 2**.

	**Audience Response**	
	**No Response *M (SD)***	**Laughter *M (SD)***
**AUTONOMY SCENARIOS**		
Scenario 1	3.91 (1.83)^a^	4.86 (1.61)^b^
Scenario 2	4.13 (1.70)^†^	4.67 (1.76)^†^
Scenario 3	3.02 (1.57)^a^	3.71 (1.68)^b^
**SOCIAL-RELATIONAL SCENARIOS**		
Scenario 4	2.65 (1.61)^a^	3.21 (1.69)^a^
Scenario 5	3.12 (1.53)^a^	3.13 (1.69)^a^
Scenario 6	4.00 (1.62)^a^	3.83 (1.82)^a^

*Means in one row with different superscripts differ at least at p < 0.05*.

† *Indicates p < 0.08*.

#### Social-relational scenarios

A similar ANCOVA was conducted for the social-relational scenarios. There was no effect for Audience Response on humiliation, *F*_(1, 96)_ = 0.27, *p* = 0.602, $\eta_{p}^{2}$ = 0.003^8^. Participants reported almost the same amount of humiliation when the audience laughed after the insult (*M* = 3.39, *SD* = 1.43) as when there was no audience response (*M* = 3.23, *SD* = 1.13). Gender was not a significant covariate, *F*_(1, 96)_ = 2.92, *p* = 0.091, $\eta_{p}^{2}$ = 0.030.

Again, we also analyzed the effect of Audience Response for each scenario separately (for means and standard deviations see Table 1). A MANCOVA with Audience Response (No Response vs. Laughter) as between-subjects factor, Gender as a covariate and reported humiliation for scenarios 4, 5, and 6 (see Appendix A) as dependent variables showed no overall main effect, Wilks' Lambda = 0.953, *F*_(3, 93)_ = 1.52, *p* = 0.215, $\eta_{p}^{2}$ = 0.047, nor univariate effects for scenario 4, *F*_(1, 95)_ = 2.58, *p* = 0.112, $\eta_{p}^{2}$ = 0.026, scenario 5, *F*_(1, 95)_ = 0.01, *p* = 0.928, $\eta_{p}^{2}$ = 0.000, or scenario 6, *F*_(1, 95)_ = 0.29, *p* = 0.593, $\eta_{p}^{2}$ = 0.003.

### Discussion

Study 2 first of all showed that participants reported more humiliation when a public insult described in a scenario concerned a threat to autonomous self-related values than when it concerned a threat to social-relational self-related values. Participants in this study also rated autonomous values as more important—and thus more central to their self-image— than social-relational values. This provides evidence for the idea that when a humiliating insult concerns a threat to more central aspects of the self, humiliation is experienced more strongly than when the insult is targeted at less central aspects of the self (Hypothesis 3).

In addition, Study 2 replicated the results from Study 1 and found that the presence of a laughing audience in descriptions of a public insult intensified reported humiliation, but this was only true for insults targeted at autonomous values and not for insults targeted at social-relational values. Importantly, because the method we used did not allow us to compare the effect of audience laughter for both types of value threats in one analysis, we cannot say anything about a possible difference between a laughter effect for the two types of threat; we can merely conclude that there was an effect of laughter for the autonomy values, but not for the social-relational values. This could indicate that—even though threats to all these values evoke considerably strong reports of humiliation—the negative response of audience laughter matters in particular when stable, positive personality characteristics, that is, central aspects of the self are threatened.

## General discussion

Humiliation is often described as an extremely negative, very intense emotion that is stuck in one's memory much more strongly than many other negative emotions. The current research focused on two explanations for the intense character of this emotion: negative audience behavior and the target of the humiliative act. In study 1, we found evidence for the hypothesis that audience laughter after a humiliating insult leads to stronger feelings of humiliation than when there is no such response. Although previous research emphasized the presence of an audience showing hostile intent as an antecedent of humiliation, this is, to our knowledge, the first study that shows a direct causal relationship between (imagined) audience laughter and reported humiliation. Study 2 provided evidence for the idea that humiliation after a public insult is experienced as more intense when the insult concerns a threat to more central (i.e., autonomous) self-related values than when the insult concerns a threat to less central (i.e., social-relational) self-related values. Furthermore, this study showed that audience laughter only intensified humiliation after a threat to the autonomous self and not after a threat to the social-relational self.

Importantly, what is considered one's central self may depend on the social and cultural context. The notion of a stable and agentic self-construal has been shown to be more crucial in Western individualistic cultures compared to collectivistic cultures, where social and situational flexibility of the self is considered more focal (Markus and Kitayama, 1991; Cross et al., 2011). Thus, one could argue that (the effect of a laughing audience on) humiliation is especially strong when the autonomous self is threatened, but only in individualistic cultures. In more collectivistic cultures, humiliation may be felt stronger, and audience laughter may have an effect in particular when it concerns a threat to the social-relational self. Indeed, there is evidence for cultural differences in antecedents of certain emotions, for example shame and anger (e.g., Markus and Kitayama, 1991; Rodriguez Mosquera et al., 2002), and in the importance of situations eliciting emotions (e.g., Markus and Kitayama, 1991; Mesquita, 2001).

However, we should be careful with drawing strong conclusions on the role of cultural values in the present data. Although, we found that participants in Study 2 scored higher on autonomous values than on social-relational values, this difference was not very large. Moreover, we did not find evidence that participants believed that social-relational values were *not* important to them, as the mean score on these values was above the midpoint. This also reflects the idea that we cannot simply divide the world into two parts, each endorsing different values (autonomy vs. social-relational). Importantly, our sample was relatively young, highly educated, and from an industrialized country and multicultural city. Thus, the findings could reflect the endorsement of a more “globalized” pattern of cultural values. Therefore, we should be careful to generalize these results to other populations (e.g., lower educated or older participants).

Future studies should compare different humiliating episodes between people from different cultures. Besides taking into account the individualistic-collectivistic dimension in such research, it would also be relevant to study other cultural dimensions with regard to humiliation, such as power distance. For example, people from a culture with a large power distance may be more accepting of inequalities and therefore feel less humiliated after a humiliation-evoking incident than when the same incident would happen in a culture with a small power distance.

Another interesting option would be to compare different humiliating scenarios among people from honor, dignity, and face cultures. For example, humiliation seems a more prevalent part of emotional life in honor cultures (e.g., Miller, 1993; Rodriguez Mosquera et al., 2002), because of the stronger emphasis on one's reputation and social status (Nisbett and Cohen, 1996). Some recent findings on national humiliation (Doosje et al., manuscript submitted for publication) indicated that people from Albania (an honor culture), Hong Kong and India (face-keeping cultures) reported more national humiliation after reading scenarios in which their nation was degraded than people from the Netherlands (a dignity culture).

In Study 1, we found an unexpected but important null-result with regard to social support. Whereas a negative audience response intensifies reports of humiliation, helping behavior after the humiliating incident does not seem to reduce reported humiliation. An explanation for this finding may partly reside in the “dark” side of social support. Although helping is generally regarded as positive and prosocial, it can also be interpreted as asserting dominance over an individual or a group (e.g., Nadler, 2002) and producing status differences between helper and recipient (e.g., Dixon et al., 2012). Receiving help and being in need of help can make people feel dependent on and inferior to the helper. This can in turn lower their self-esteem (e.g., Nadler and Fischer, 1986) by keeping an unequal power relation in place, rendering the receiver unable to take control over the situation. This is relevant because a lack of control seems to be an important feature of humiliation. The humiliating incident itself probably causes a lowered self-esteem in the victim which may be further reduced, or at least not restored, by the support, and as such does not diminish feelings of humiliation. In this regard, the identity of the helper seems important as well. For example, if support is offered by a person higher in status than the victim it may increase feelings of humiliation because it confirms the victim's dependency. Similarly, support offered by a stranger may not have the intended effect, but merely emphasizes the publicity of the event. If, however, support is offered by a (trusted) peer or friend of equal status the result could be a decrease in experienced humiliation. Thus, for future research, it seems crucial to take into account the nature and source of the support.

## Limitations and future directions

A limitation of the present research is the use of scenarios. Although, this is an often used and valid method for measuring *imagined* or *expected* emotions, it is difficult to determine whether people really *feel* certain emotions as a result of reading such a scenario. Nevertheless, we think that people's concepts of humiliation provide us with a good gauge of how humiliation may be caused and experienced in reality. Moreover, research by Otten and Jonas (2014) showed that scenarios designed to induce either humiliation, shame, anger, or happiness evoked different patterns of brain activity (using EEG) in participants. Although this does not prove that the “real” emotion is indeed felt, the fact that these scenarios differently affected the brain adds to their validity in distinguishing these emotions. Still, we think it is important to develop other methods to examine the causes and effects of humiliation, such as inducing feelings of humiliation in the laboratory, in the presence of an audience. This approach, although ethically challenging, allows for a more controlled examination of antecedents and consequences of humiliation.

In addition, we consider it important to examine the relation between humiliation, shame, and anger in different (cultural) contexts. We already know that humiliation is related to shame on the one hand and anger on the other, but these relationships may differ depending on contextual aspects, such as whether the episode concerns individual or group-based humiliation, and the extent to which the humiliating event is appraised as unfair. More unfairness is likely related to more anger as part of the humiliating experience.

Fear might be another important emotion that could be related to humiliation, especially when humiliation is experienced more frequently. One of the consequences of repeated episodes of humiliation may be the development of social anxiety, or more in particular, a strong fear of situations involving an audience. Such anxiety may eventually turn into *gelotophobia*, a fear of being laughed at (e.g., Titze, 2009), and prevent people from interacting with others in satisfying ways. Because humiliation is such an intense emotion, even a single humiliating event may have a strong impact on the victim. However, this impact also likely depends on people's (trait) self-esteem. Self-esteem functions as a buffer against anxiety (Pyszczynski et al., 2004). Thus, people who have higher self-esteem could be more resilient to humiliation and the effect of a humiliating act may be less detrimental than for people with low self-esteem who may be more humiliation prone.

Not only fear, but also anger may become more strongly associated with humiliation over time, and thoughts about revenge could develop as a result of enhanced opportunities to ruminate about the humiliating episode. Moreover, the strong hypothesized link between humiliation and revenge (e.g., Lickel, 2012) may become apparent only after repeated incidents of humiliation. Being (or perceiving to be) frequently humiliated over a longer period of time, creates the opportunity for the victim to develop feelings of rage and revenge instead of, or next to, shame and fear. This process of rumination leading to revenge may also be an important element of group-based humiliation, a phenomenon that received much theoretical attention but is lacking empirical evidence.

Another important avenue for future studies is that of humiliation in the context of social media. Humiliation can happen online, for example in the form of cyberbullying. Importantly, cyberbullying differs from traditional (offline) bullying in that in cyberbullying humiliating texts, images or videos of the victim can reach a very large (even unlimited) audience in a very short time (e.g., Heirman and Walrave, 2015). Thus, the impact of an audience on feelings of humiliation we found in the current research may play an even larger role when humiliation takes place on social media.

Finally, we wish to note that studying humiliation is difficult, particularly because of its aversive character rendering people unwilling to think and talk about it. This means that there are ethical issues inherent to the study of humiliation. This is not only the case when asking people to report on their humiliating experiences, but also when one attempts to experimentally study humiliation. One can only manipulate low levels of humiliation in the laboratory (see e.g., Mann et al., 2016), which seems paradoxical as we argued that humiliation is very intense by definition. The study of contextual and cultural factors contributing to the intensity of humiliation is very relevant, however, because humiliation seems to be an important motivator for aggression and violence on the one hand and shame and social phobia on the other, so it is important to find out in what specific ways. In the present studies, we have shown that a humiliating experience becomes even more intense when central aspects of one's identity are attacked and when one is confronted with a laughing derogatory audience.

## Ethics statement

Study 1 did not require ethical approval according to the national and institutional guidelines at the time of the study commencement, consent for this study was considered implicit by the return of the questionnaire. Study 2 was reviewed and approved by the Faculty of Ethics Review Board of the UVA, and written informed consent was obtained from all participants.

## Author contributions

The studies were designed by all authors. Data were collected and analyzed by LM and AL. The article was written by LM and ARF, BD, and AHF provided valuable comments on the manuscript.

## Funding

This research was partly funded by a grant from the European Union awarded to the University of Amsterdam (amongst others) for their work on the European FP-7 project SAFIRE (Project Reference: 241744).

### Conflict of interest statement

The authors declare that the research was conducted in the absence of any commercial or financial relationships that could be construed as a potential conflict of interest.

## Footnotes

^1^We would like to thank one of the reviewers for pointing this out.

^2^Because (a) they did not participate in a serious manner (*n* = 8); (b) their responses were incomplete (*n* = 5); or (c) they scored too high on the item “happiness” (4 or 5 on a five-point scale), which was intended as a control variable to test the effectiveness of the scenario (*n* = 4).

^3^In addition, we asked people whether they would experience shame, anger, and contempt. Shame was positively related to humiliation (*r* = 0.58, *p* < 0.001), as was anger (*r* = 0.30, *p* = 0.001). Contempt was unrelated to humiliation (*r* = 0.03, *p* = 0.771). We also included the emotion label “happiness” as a control item. If participants scored high on this item (4 or 5), their data were excluded from the analyses, because in these instances we considered the manipulation of humiliation as failed. Happiness was negatively related to humiliation (*r* = −0.23, *p* = 0.012).

^4^There was also a significant main effect of Audience Response on shame, *F*_(1, 106)_ = 5.99, *p* = 0.016, $\eta_{p}^{2}=$ 0.053, and a marginal effect of Audience Response on anger, *F*_(1, 107)_ = 3.81, *p* = 0.054, $\eta_{p}^{2}=$ 0.034, indicating stronger reports of these emotions when the audience laughed than when there was no audience response. Audience Laughter did not affect reports of contempt or happiness. In addition, there was an effect of Social Support on anger, *F*_(1, 107)_ = 17.33, *p* < 0.001, $\eta_{p}^{2}=$ 0.139, and on happiness, *F*_(1, 107)_ = 25.04, *p* < 0.001, $\eta_{p}^{2}=$ 0.19 (but not on shame or contempt). Participants reported less anger and more happiness when support was offered than when no support was offered after the humiliating episode. There were no interactions between Audience Response and Social Support on any of the emotions.

^5^Again, we also asked people whether they would experience shame and anger. Shame was positively related to humiliation for all scenarios; correlations ranged from *r* = 0.41 to *r* = 0.78 (all *p*'s < 0.001). Anger was unrelated to humiliation for scenario 3 (see Appendix), but it was positively related to humiliation for the other scenarios; correlations ranged from *r* = 0.18 to *r* = 0.47 (all *p*'s ≤ 0.07). In addition, we measured sadness, which was positively related to humiliation for all scenarios; correlations ranged from *r* = 0.39 to *r* = 0.68 (all *p*'s < 0.001).

^6^For the autonomy scenarios, correlations for humiliation ranged from: *r* = 0.48 to *r* = 0.49 (all *p'*s < 0.001). For the social-relational scenarios, correlations for humiliation ranged from: *r* = 0.33 to *r* = 0.46 (all *p'*s ≤ 0.001).

^7,8^There were no effects of Audience Response on shame, anger, or sadness.

## Appendix A

### Scenarios study 2

Two different versions of the questionnaire were created to counterbalance the order of audience laughter vs. no audience response. In version 1, the audience laughed in the social-relational scenarios, and in version 2, the audience laughed in the autonomy scenarios.

#### Autonomy scenarios:

1. You are participating in a discussion on politics, hosting people with different backgrounds. At a certain moment the discussion turns to a sensitive subject for some people. The discussion leader happens to be aware that you know a lot about this subject and he asks you a question about it. When you hesitate a little to answer his question, the person who sits next to you says in a sneering tone: “If you are not even able to give an honest and open opinion, then what are you doing here?” (Some of the other discussants start to laugh.)
2. You are in a café talking to some friends. The conversation is about politics. You make a comment about politician x, saying that you think he did some good things for his voters. All your friends disagree with you on this. You didn't expect this and quickly add: “Yeah, you are right, he did made a lot of mistakes.” Then, one of your friends says: “You really don't have your own opinion.” (The rest of the group starts to laugh.)
3. You are joining a workgroup in which you are supposed to make an assignment and present it afterwards with other students. You thought of a nice idea for this assignment and start to explain your idea enthusiastically to the rest of the group. Suddenly, another group member starts to laugh out loud: “Well, that is not very original, I heard them propose the same idea in the other group. I thought you would come up with something more exiting!” (The rest of the group also starts to laugh.)

#### Social-relational scenarios:

4. It is Friday morning and you have a lot of work to do. However, it is your aunt's birthday today and she will give a big party that starts in the afternoon. You really want to finish some work before you leave and at 17:30 you clean your desk to go to the party. You explain to your colleagues that you are in a hurry because the party has already started. Then, one of your colleagues gives you a serious look and says: “How unkind of you to arrive that late at your aunt's birthday, you could at least have helped her with preparations.” (Some other colleagues start to laugh.)
5. You are telling a story to your colleagues that you find very funny. It is about your very old uncle whom you were visiting a while ago in the care home where he lives. Your uncle asked you every 5 minutes what kind of job you have and kept on telling you the story about the boat-tour he made last week. You have to laugh very hard about this. Suddenly, a colleague looks at you and says: “You really have no respect for elderly people, how would you feel if people didn't take you seriously anymore at that age!” (Your other colleagues start to laugh.)
6. You are sitting in a tram full of people, when a disabled woman with crutches enters the vehicle. Nobody stands up for the woman, neither do you. This is not because you don't want to but actually you don't feel well and you're sure that someone else will offer his seat to her. Then an older gentleman behind you offers his seat to the woman. He looks at you disdainful and says: “Can't you even show some respect to somebody else!” (Some of the other passengers start to laugh.)

## References

[B1] Collazzoni A. Capanna C. Bustini M. Marucci C. Prescenzo S. Ragusa M.. (2015). A comparison of humiliation measurement in a depressive versus non-clinical sample: a possible clinical utility. J. Clin. Psychol. 71, 1218–1224. 10.1002/jclp.22212 26275166

[B2] Collazzoni A. Capanna C. Bustini M. Stratta P. Ragusa M. Marino A.. (2014). Humiliation and interpersonal sensitivity in depression. J. Affect. Disord. 167, 224–227. 10.1016/j.jad.2014.06.008 24995891

[B3] Combs D. J. Y. Campbell G. Jackson M. Smith R. H. (2010). Exploring the consequences of humiliating a moral transgressor. Basic Appl. Soc. Psychol. 32, 128–143. 10.1080/01973531003738379

[B4] Cowie H. Hutson N. (2005). Peer support: a strategy to help bystanders challenge school bullying. Pastoral Care, 23, 40–44. 10.1111/j.0264-3944.2005.00331.x

[B5] Cross S. E. Harding E. E. Gercek-Swing B. (2011). The What, how, why and where of self-construal. Pers. Soc. Psychol. Rev. 15, 142–179. 10.1177/1088868310373752 20716643

[B6] Dixon J. Levine M. Reicher S. Durrheim K. (2012). Beyond prejudice: are negative evaluations the problem and is getting us to like one another more the solution? Behav. Brain Sci. 35, 411–425. 10.1017/S0140525X11002214 23164194

[B7] Ekman P. Friesen W. V. (1986). A new pan-cultural facial expression of emotion. Motiv. Emot. 10, 159–168. 10.1007/BF00992253

[B8] Elison J. Harter S. (2007). Humiliation: causes, correlates, and consequences, in The Self-Conscious Emotions: Theory and Research, eds Tracy J. L. Robins R. W. Tangney J. P.(New York, NY: Guilford Press), 310–329.

[B9] Farmer A. E. McGuffin P. (2003). Humiliation, loss and other types of life events and difficulties: a comparison of depressed subjects, healthy controls and their siblings. Psychol. Med. 33, 1169–1175. 10.1017/S0033291703008419 14580071

[B10] Fernández S. Saguy T. Halperin E. (2015). The Paradox of Humiliation: the acceptance of an unjust devaluation of the self. Pers. Soc. Psychol. Bull. 41, 976–988. 10.1177/0146167215586195 25987554

[B11] Fischer A. H. Evers C. (2013). The social basis of emotion in men and women, in The SAGE Handbook of Gender and Psychology, eds Ryan M. K. Branscombe N. R.(London: Sage Publications), 183–198.

[B12] Fischer A. H. Giner-Sorolla R. (2016). Contempt: derogating others while keeping calm. Emot. Rev. 8, 346–357. 10.1177/1754073915610439

[B13] Gilbert P. (1997). The evolution of social attractiveness and its role in shame, humiliation, guilt and therapy. Br. J. Med. Psychol. 70, 113–147. 10.1111/j.2044-8341.1997.tb01893.x 9210990

[B14] Harter S. (2012). Self-conscious emotions in The Construction of the Self. Developmental and Sociocultural Foundations, 2nd Edn., ed Harter S.(New York, NY: Guilford Press), 194–233.

[B15] Hartling L. (2007). Humiliation: Real pain, a pathway to violence. RBSE 6, 276–290. Available online at: http://www.humiliationstudies.org/documents/hartling/HartlingNY05meetingRT2.pdf

[B16] Hartling L. M. Luchetta T. (1999). Humiliation: assessing the impact of derision, degradation, and debasement. J. Prim. Prev. 19, 259–278. 10.1023/A:1022622422521

[B17] Heirman W. Walrave M. (2015). Assessing concerns and issues about the mediation of technology in cyberbullying. Cyberpsychol. J. Psychosoc. Res. Cyberspace 2:1 Available online at: https://journals.muni.cz/cyberpsychology/article/view/4214/3256

[B18] Jackson M. A. (2000). Distinguishing Shame and Humiliation. Doctoral. dissertation, Retrieved from Dissertations and Theses database. (UMI No. 731880811)

[B19] Kendler K. S. Hettema J. M. Butera F. Gardner C. O. Prescott C. A. (2003). Life event dimensions of loss, humiliation, entrapment, and danger in the prediction of onsets of major depression and generalized anxiety. Arch. Gen. Psychiatry 60, 789–796. 10.1001/archpsyc.60.8.789 12912762

[B20] Klein D. C. (1991). The humiliation dynamic: an overview. J. Prim. Prev. 12, 93–121. 10.1007/BF02015214 24258218

[B21] Leary M. R. Kowalski R. M. Smith L. Phillips S. (2003). Teasing, rejection, and violence: case studies of the school shootings. Aggress. Behav. 29, 202–214. 10.1002/ab.10061

[B22] Leidner B. Sheikh H. Ginges J. (2012). Affective dimensions of intergroup humiliation. PLoS ONE 7:e46375. 10.1371/journal.pone.0046375 23029499 PMC3460861

[B23] Lewis H. B. (1971). Shame and Guilt in Neurosis. New York, NY: International University Press, Inc.

[B24] Lewis M. (1992). Shame. The Exposed Self. New York, NY: The Free Press.

[B25] Lewis M. (1995). Self-conscious emotions. Am. Sci. 83, 68–78.

[B26] Lickel B. (2012). Retribution and revenge, in The Oxford Handbook of Intergroup Conflict, ed Tropp L. R.(New York, NY: Oxford University Press), 89–105.

[B27] Lindner E. G. (2009). Genocide, humiliation, and inferiority. An interdisciplinary perspective, in Genocides by the Oppressed: Subaltern Genocide in Theory and Practice, eds Robins N. A. Jones A.(Bloomington, IN: Indiana University Press), 138–159.

[B28] Mann L. Feddes A. R. Doosje B. Fischer A. H. (2016). Withdraw or affiliate? The role of humiliation during initiation rituals. Cogn. Emot. 30, 80–100. 10.1080/02699931.2015.1050358 26220561

[B29] Markus H. R. Kitayama S. (1991). Culture and the self: implications for cognition, emotion, and motivation. Psychol. Rev. 98, 224–253. 10.1037/0033-295X.98.2.224

[B30] Mesquita B. (2001). Emotions in collectivist and individualist contexts. J. Pers. Soc. Psychol. 80, 68–74. 10.1037/0022-3514.80.1.68 11195892

[B31] Miller W. I. (1993). Humiliation and Other Essays on Honor, Social Discomfort, and Violence. Ithaca, NY: Cornell University Press.

[B32] Nadler A. (2002). Inter-group helping relations as power relations: maintaining or challenging social dominance between groups through helping. J. Soc. Issues 58, 487–502. 10.1111/1540-4560.00272

[B33] Nadler A. Fischer J. D. (1986). The role of threat to self-esteem and perceived control in recipient reaction to help: theory development and empirical validation, in Advances in Experimental Social Psychology, ed Berkowitz L.(New York, NY: Academic Press), 81–121.

[B34] Naylor P. Cowie H. (1999). The effectiveness of peer support systems in challenging school bullying: the perspectives and experiences of teachers and pupils. J. Adolesc. 22, 467–479. 10.1006/jado.1999.0241 10469511

[B35] Niedenthal P. M. Mermillod M. Maringer M. Hess U. (2010). The Simulation of Smiles (SIMS) model: embodied simulation and the meaning of facial expression. Behav. Brain Sci. 33, 417–433. 10.1017/S0140525X10000865 21211115

[B36] Nisbett R. E. Cohen D. (1996). Culture of honor: The psychology of Violence in the South. Boulder, CO: Westview Press.

[B37] Otten M. Jonas K. J. (2014). Humiliation as an intense emotional experience: evidence from the electro-encephalogram. Soc. Neurosci. 9, 23–35. 10.1080/17470919.2013.855660 24215103

[B38] Pyszczynski T. Greenberg J. Solomon S. Arndt J. Schimel J. (2004). Why do people need self-esteem? A theoretical and empirical review. Psychol. Bull. 130, 435–468. 10.1037/0033-2909.130.3.435 15122930

[B39] Rodriguez Mosquera P. M. Manstead A. S. R. Fischer A. H. (2002). The role of honour concerns in emotional reactions to offences. Cogn. Emot. 16, 143–163. 10.1080/02699930143000167

[B40] Ruch W. Proyer R. T. (2008). The fear of being laughed at: individual and group differences in gelotophobia. Humor 21, 47–67. 10.1515/HUMOR.2008.002

[B41] Sauter D. A. Eisner F. Ekman P. Scott S. K. (2010). Cross-cultural recognition of basic emotions through nonverbal emotional vocalizations. Proc. Natl. Acad. Sci. U.S.A. 107, 2408–2412. 10.1073/pnas.0908239106 20133790 PMC2823868

[B42] Schwartz S. H. (2006). A theory of cultural value orientations: explication and applications. Comp. Sociol. 5, 137–180. 10.1163/156913306778667357

[B43] Schwartz S. H. Bilsky W. (1990). Toward a theory of the universal content and structure of values: extensions and cross-cultural replications. J. Pers. Soc. Psychol. 58, 878–891. 10.1037/0022-3514.58.5.878

[B44] Schwartz S. H. Rubel T. (2005). Sex differences in value priorities: cross-cultural and multimethod studies. J. Pers. Soc. Psychol. 89:1010. 10.1037/0022-3514.89.6.1010 16393031

[B45] Scott S. K. Lavan N. Chen S. McGettigan C. (2014). The social life of laughter. Trends Cogn. Sci. 18, 618–620. 10.1016/j.tics.2014.09.002 25439499 PMC4255480

[B46] Smith R. H. Webster J. M. Parrott W. G. Eyre H. L. (2002). The role of public exposure in moral and nonmoral shame and guilt. J. Pers. Soc. Psychol. 83, 138–159. 10.1037/0022-3514.83.1.138 12088123

[B47] Titze M. (2009). Gelotophobia: the fear of being laughed at. Humor. Int. J. Humor Res. 22, 27–48. 10.1515/humr.2009.002

[B48] Torres W. J. Bergner R. M. (2010). Humiliation: its nature and consequences. J. Am. Acad. Psychiatry Law 38, 195–204. Available online: http://sdp.org/publications/papers/Humiliation%20It%27s%20Nature%20and%20Consequences.pdf 20542938

[B49] van Dijk W. W. Ouwerkerk J. W. Goslinga S. Nieweg M. Gallucci M. (2006). When people fall from grace: reconsidering the role of envy in schadenfreude. Emotion 6, 156–160. 10.1037/1528-3542.6.1.156 16637759

[B50] Veldhuis T. M. Gordijn E. H. Veenstra R. Lindenberg S. (2014). Vicarious group-based rejection: creating a potentially dangerous mix of humiliation, powerlessness, and anger. PLoS ONE 9:e95421. 10.1371/journal.pone.0095421 24759901 PMC3997388

[B51] Wagner H. L. (2000). The accessibility of the term “contempt” and the meaning of the unilateral lip curl. Cogn. Emot. 14, 689–710. 10.1080/02699930050117675

[B52] Walker J. Knauer V. (2011). Humiliation, self-esteem and violence. J. Forens. Psychiatry Psychol. 22, 724–741. 10.1080/14789949.2011.617542

[B53] West L. (2014). Why Humiliation is the Most Intense Human Emotion [Web log post]. Available online at: http://jezebel.com/why-humiliation-is-the-most-intense-human-emotion-1572014449 (Accessed May 7, 2014).

[B54] Zavaleta Reyles D. (2007). The ability to go about without shame: a proposal for internationally comparable indicators of shame. Oxf. Dev. Stud. 35, 405–430. 10.1080/13600810701701905

